# SKIP (Supporting Kids with diabetes In Physical activity): Feasibility of a randomised controlled trial of a digital intervention for 9-12 year olds with type 1 diabetes mellitus

**DOI:** 10.1186/s12889-019-6697-1

**Published:** 2019-04-03

**Authors:** Emily Knox, Cris Glazebrook, Tabitha Randell, Paul Leighton, Boliang Guo, James Greening, E. Bethan Davies, Lori Amor, Holly Blake

**Affiliations:** 1 University of Nottingham, School of Health Sciences, Nottingham, UK; 2 University of Nottingham, School of Medicine, Nottingham, UK; 3 Nottingham University Hospitals NHS Trust, Nottingham, UK; 4 University Hospitals of Leicester NHS Trust, Leicester, UK; 5 NIHR MindTech MedTech Co-operative, Institute of Mental Health, University of Nottingham, Triumph Road, Nottingham, UK; 6 NIHR Nottingham Biomedical Research Centre, Nottingham, UK

**Keywords:** Type 1 diabetes mellitus, Physical activity, Children, Health, Feasibility, Website, Intervention

## Abstract

**Background:**

Physical activity and self-monitoring are important for children with type 1 diabetes mellitus (T1DM) but it is unclear whether interventions delivered online are feasible, acceptable to patients and efficacious. The aim was to assess the feasibility and acceptability of an internet-based physical activity and self-monitoring programme for children with T1DM, and of a randomised controlled trial (RCT) to evaluate efficacy.

**Methods:**

A total of 49 children aged 9-12 with T1DM were randomly assigned to usual care only or to an interactive intervention group combining a website (STAK-D) and a PolarActive activity watch (PAW; Polar Electro (UK) Ltd.), alongside usual care. Participants completed self-report measures on their health, self-efficacy and physical activity at baseline (T0), eight weeks (T1) and six months (T2). They also wore a PAW to measure physical activity for one week at the end of T0, T1 and T2. Intervention participants were interviewed about their experiences at T2. Explanatory variables were examined using multi-level modelling and examination of change scores, 95% confidence intervals and p-values with alpha set at 0.95. Descriptive analysis was undertaken of the ‘end-of-study questionnaire’. Qualitative analysis followed a framework approach.

**Results:**

Completion rates for all self-report items and objective physical activity data were above 85% for the majority of measures. HbA1c data was obtained for 100% of participants, although complete clinical data was available for 63.3% to 63.5% of participants at each data collection time-point. Recruitment and data collection processes were reported to be acceptable to participants and healthcare professionals. Self-reported sedentary behaviour (-2.28, *p*=0.04, 95% CI=-4.40, -0.16; p = 0.04; *d*_*ppc2*_ = 0.72) and parent-reported physical health of the child (6.15, p=0.01, 95%CI=1.75, 10.55; p = 0.01; *d*_*ppc2*_ = 0.75) improved at eight weeks in the intervention group.

**Conclusions:**

The trial design was feasible and acceptable to participants and healthcare providers. Intervention engagement was low and technical challenges were evident in both online and activity watch elements, although enjoyment was high among users. Reported outcome improvements were observed at 8 weeks but were not sustained.

**Trial registration:**

ISRCTN 48994721 (prospectively registered). Date of registration: 28.09.2016.

**Electronic supplementary material:**

The online version of this article (10.1186/s12889-019-6697-1) contains supplementary material, which is available to authorized users.

## Background

Type 1 diabetes mellitus (T1DM) is increasing in prevalence amongst children in the UK [1]. It is challenging for children with the condition to take responsibility over self-management activities, with parents often assuming this role [2]. While parental involvement and oversight is important, particularly with younger children, too much referral of responsibility to parents may hinder the child’s competence to effectively control their condition into adulthood. One behaviour that is important in the control of T1DM is physical activity [3]. However, children with diabetes often fail to meet physical activity recommendations [4, 5]. Health care provision should therefore include components that target the diabetic child’s ability, confidence and motivation to self-monitor and safely engage in more physical activity.

To be useful in healthcare settings, interventions need to be adaptable, cost-effective and acceptable to healthcare professionals. Activity-based interventions within this population typically require supervision and attendance at specialist facilities, which limits acceptability and economic sustainability [6]. Children engage routinely with various forms of technology [7]. Further, digital technology is a medium through which children may process information even more confidently than their elders due to the generational gap. Its use has shown some promise as a potentially efficacious and cost-efficient tool in the management of chronic conditions in children [8]. However, evidence is still lacking regarding the feasibility and efficacy of technology-based interventions and the research processes used to evaluate them. A recent systematic review examined the role of technology in the self-management of type 1 diabetes mellitus (T1DM) among children and young people [9]. The review included interventions targeting key diabetes self-management behaviours (self-management of blood glucose, insulin administration, physical activity and dietary behaviours) and prerequisites (psychological outcomes and HbA1c) as highlighted in the UK guidelines of the National Institute for Health and Care Excellence (NICE) for management of T1DM among children and young people. Technology-based interventions showed positive effects for some self-management behaviours (such as self-monitoring of blood glucose) although the impact on physical activity was unclear due to lack of evidence [9]. The aim of the present study therefore, was to assess the feasibility and acceptability of an internet-based physical activity and self-monitoring programme for children with T1DM and a randomised controlled trial (RCT) designed to evaluate it.

## Methods

Detailed methods have been published elsewhere including full details of the demographic and clinical measures taken [10]. This article reports on the acceptability and feasibility of the study design and intervention, and preliminary indications of efficacy.

### Study design and methods

The SKIP study (Supporting Kids with Diabetes in Physical Activity) was a randomised controlled trial (RCT) testing an online physical activity and self-monitoring intervention called STAK-D (Steps to Active Kids with Diabetes [11]). Feasibility of recruitment and data collection processes of the SKIP study were assessed using a mixed-methods design [10]. CONSORT reporting is shown in Additional file 1. The study was undertaken between October 2016 and July 2017 in two East Midlands NHS university hospitals in the UK (Nottingham University Hospitals NHS Trust [NUH]; University Hospitals of Leicester NHS Trust [UHL]).

We aimed to recruit 50 patients (25 from each site), in order to estimate preliminary efficacy of the intervention and inform decisions for a future RCT [12]. Eligible participants were aged 9-12 years at study start; diagnosed with T1DM for at least three months and without consultant concern for engagement in physical activity. The inclusion criteria did not change throughout the study.

### Recruitment and randomisation

**Fig. 1:**
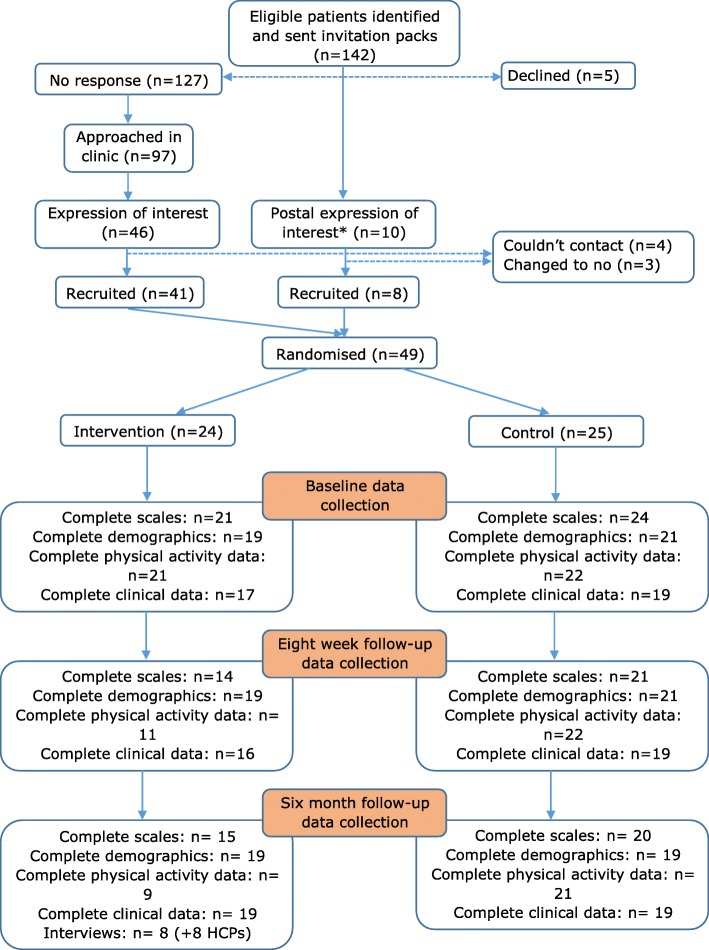
Flow of participants in SKIP study

Study flow is provided in Fig. 1. Information packs were sent from the clinic to all eligible patients. Patients who returned the enclosed expression of interest slip were then contacted by the project researcher. Patients who did not return the expression of interest slip were then approached during their next regular clinic appointment and invited to participate. Written informed consent and assent were taken from parents and children respectively, and the ethical principles of the Declaration of Helsinki were adhered to. Ethical approval was obtained from ‘East Midlands - Nottingham 2 Research Ethics Committee’ in June 2016 (Ref: 16/EM/0223). Participants completed questionnaires at baseline (T0), eight weeks (T1) and six months (T2) following recruitment. They were also asked to wear a Polar Active activity watch (PAW; Polar Electro (UK) Ltd) for one week at T0, T1 and T2 to measure steps and minutes of light, moderate and vigorous physical activity each day. Each time-point therefore required two visits to participants in order to administer and collect objective physical activity data. The first author and project researcher carried out randomisation processes and conducted all visits to recruit participants, administer intervention processes and collect data [10].

Participants completed baseline questionnaires before being randomised to intervention or control using a simple 1:1 allocation ratio via a secure online service. Blinding was not possible given the nature of the intervention.

### The STAK-D intervention

Intervention and control participants continued with their usual care. Usual care was measured at baseline and following the completion of T1 and T2. The lead consultant at each participating site completed a questionnaire to define usual care, which was adapted from the framework described by Erlen and colleagues [13]. Responses to individual items were summed to produce an overall usual care score. Intervention participants received access to the STAK-D website which has been described elsewhere [10]. All 24 intervention participants were given website login credentials, a website information package and an introductory session with a project researcher. STAK-D combined behaviour change techniques including physical activity goal setting, feedback and increasing knowledge with the aim of increasing participant’s self-efficacy for diabetes self-management (e.g. confidence around management of physical activity alongside diet, and regular blood glucose self-monitoring). The intervention group were also provided with a PAW (Polar Electro (UK) Ltd.) and associated guidance on use, which they were encouraged to wear for the duration of the six-month study.

### Primary outcomes: Feasibility measures

Primary measures addressed the feasibility, acceptability, fidelity and contextual influences of the delivery of SKIP (Table 1). Data were collected on rates of recruitment, consent, retention and adverse events. Reasons for non-participation and withdrawal were collected, where possible. Objective physical activity data were included if the child wore the watch for at least 600 minutes a day on three days [14]. Adherence to STAK-D was evaluated by examining website logins and downloads. Parents and children independently completed a five-item burden questionnaire at T0, T1 and T2, which recorded perceived time taken to complete the questionnaire, comprehension and difficulty of the questionnaire and whether help was needed to complete the questionnaire items. We determined the acceptability and feasibility of completing questionnaires and wearing a wrist-worn activity monitor at >70% and >85% completion rates, respectively [15]. Fidelity of project delivery was also considered.

**Table 1:** Indicators used to evaluate feasibility, acceptability, fidelity and contextual influences

Process evaluation tasks	How this was enacted
*Feasibility.* Exposure to the intervention/elements of the intervention	Number of participants given login credentials, website information package and introductory session Problems with watch syncing software Interviews with participants/parents
*Acceptability.* Participation (describing intervention participation rates); these include measures of ‘recruitment’ to the program and ‘reach into a population’, program satisfaction	Number approached/recruited Reasons for refusal Methods of recruitment Engagement with website/website components Engagement with watch syncing software Participant/parent burden and satisfaction questionnaires Interviews with participants/parents
*Fidelity.* Delivery of the intervention, or assessing scaling-up of the intervention to larger populations	Number of prompts/activity reports sent Location and completeness of data collection Difference in delivery across sites Adaptations made to deliver the program
*Contextual influences.* Context of the intervention	Log of problems in the delivery of SKIP, problems experienced, barriers to implementation Record of ways in which SKIP was delivered differently across sites and reasons. Interviews with healthcare practitioners

At T2, control and intervention parents completed an additional end-of-study questionnaire, reporting their child’s access to technological devices relative to two time-points: prior to SKIP and at SKIP end. Intervention group parents and children also responded to three additional measures at T2 assessing enjoyment, learning and behaviour change resulting from SKIP.

Following the final follow-up (T2), eight participant-parent dyads from the intervention group and eight healthcare professionals (HCP) took part in semi-structured interviews exploring the acceptability of SKIP, randomisation process and outcome measures (see Additional file 2). Interviews were conducted by a study researcher and audio recorded with permission. Participants could choose to be interviewed over the telephone or face-to-face.

### Secondary outcomes: Between-group outcomes

Outcome measures for assessing the potential efficacy of the intervention included clinician-patient communication about physical activity, self-reported physical activity (PAQ [16]), predilection for physical activity (CSAPPA [17]), fear of hypoglycaemia (HFS parent and child versions [18]), perceived health (CHU-9D [19]) and parents’ perception of their child’s health (CHQ [20]). Glycosylated haemoglobin (HbA1c), insulin dosage and body composition (body mass index calculated from directly measured height and weight) at T0, T1 and T2, were recorded from the patients clinic notes.

### Analysis

Differences between the baseline characteristics of those who completed or missed follow-ups were examined using chi-squared analysis and t-tests, as appropriate. Descriptive statistics for all between-group outcome measures are presented as means (SD) (Table 2). Treatment effects were examined using multi-level modelling and presented together with their 95% confidence intervals and p-values with alpha set at two-sided 0.05 level. Descriptive analysis was undertaken of the end-of-study questionnaire. Data were analysed using Stata version 15.1.

**Table 2:** Means and standard deviations for collected variables at baseline, eight weeks and six months according to group.

	Baseline		Eight week		Six month	
	Control	INT	Control	INT	Control	INT
Child measures						
Communication	1.28 ± 1.06	1.04 ± 1.46	0.78 ± 0.90	1.21 ± 1.05	1.10 ± 1.14	0.94 ± 1.34
PAQ physical activity score	57.48 ± 8.45	56.61 ± 8.77	57.57 ± 13.33	57.36 ± 8.65	58.05 ± 14.28	56.80 ± 9.12
PAQ sedentary score	22.48 ± 4.48	23.04 ± 3.65	22.70 ± 4.29	20.21 ± 3.73	22.29 ± 4.85	21.47 ± 3.91
Frequency of after school clubs	4.09 ± 4.34	4.09 ± 4.34	5.57 ± 5.68	5.57 ± 5.68	5.06 ± 3.36	5.06 ± 3.36
HFS worry	15.76 ± 10.47	19.35 ±13.05	12.30 ± 7.10	16.36 ±12.13	13.20 ± 12.37	12.69 ±14.83
HFS do	18.68 ± 6.10	19.04 ± 7.08	16.70 ± 5.91	19.21 ± 7.80	18.76 ± 6.08	17.94 ± 6.61
HFS total	34.44 ± 11.93	38.39± 16.94	29.00 ± 10.49	35.57± 16.04	31.60 ± 11.74	30.63± 18.70
CSAPPA adequacy	22.12 ± 4.28	22.42 ± 3.93	21.61 ± 5.32	23.36 ± 4.34	21.52 ± 5.59	23.25 ± 2.71
CSAPPA predilection	28.36 ± 4.80	27.54 ± 5.28	32.17 ± 5.77	32.39 ± 5.46	31.86 ± 5.70	32.19 ± 5.59
CSAPPA enjoyment	10.56 ± 1.53	10.42 ± 1.56	10.65 ± 1.92	10.79 ± 1.42	10.67 ± 1.88	10.94 ± 1.29
CSAPPA	65.12 ± 9.52	64.29 ± 9.29	64.43 ± 12.16	66.43± 10.27	64.05 ± 12.00	66.38 ± 8.47
CHU9D	12.72 ± 3.30	12.17 ± 3.63	12.78 ± 2.78	12.36 ± 3.15	12.71 ± 3.42	14.13 ± 5.14
HbA1c (mmol/mol)	53.50 ± 11.61	54.57 ± 9.37	53.58 ± 8.83	58.91 ± 9.05	55.65 ± 8.31	61.09 ±14.96
HbA1c (%)	7.00 ± 1.18	7.15 ± 0.85	7.05 ± 0.81	7.52 ± 0.83	7.24 ± 0.77	7.73 ± 1.38
Parent measures						
Baseline	1.28 ± 1.06	1.04 ± 1.46	0.78 ± 0.90	1.21 ± 1.05	1.10 ± 1.14	0.94 ± 1.34
Days off school	2.60 ± 9.33	1.96 ± 2.98	0.57 ± 1.20	1.00 ± 1.57	0.38 ± 1.16	1.25 ± 1.77
CHQ physical T-score	47.99 ± 9.62	44.92 ±12.16	46.30 ± 10.90	51.77 ± 6.21	47.81 ± 9.67	50.06 ± 9.76
CHQ psychological T-score	48.83 ± 11.98	44.79± 14.12	48.35 ± 11.37	48.53 ± 9.55	51.01 ± 11.75	47.61± 11.91
HFS worry	25.72 ± 15.56	29.09 ±14.91	24.91 ± 15.29	21.93± 11.18	21.33 ± 15.73	21.00 ±13.22
HFS do	20.92 ± 5.07	23.87 ± 5.94	23.35 ± 5.02	23.07 ± 6.81	21.38 ± 6.70	20.81 ± 6.86
HFS total	46.64 ± 17.89	52.96 ±19.25	48.26 ± 17.53	45.00± 15.82	42.71 ± 19.90	41.81± 16.57
HbA1c low knowledge	0.13 ± 0.56	0.12 ± 0.63	0.14 ± 0.63	0.15 ± 0.36	0.25 ± 0.90	0.12 ± 0.39
HbA1c high knowledge	2.96 ± 2.39	2.82 ± 2.69	3.07 ± 2.43	2.96 ± 2.47	3.33 ± 2.69	2.56 ± 2.94
Burden						
Clinician measures						
Communication	19.36 ± 4.32	19.42 ± 3.62	21.09 ± 3.63	21.58 ± 3.15	21.62 ± 3.23	22.59 ± 2.43
Objective physical activity						
Weekly steps	16183 ± 4717	16004±4462	16524 ± 4294	18562± 4746	16323± 4058	18215± 5755
Moderate-vigorous minutes	74.29 ± 33.09	66.18±29.17	73.29 ± 31.31	79.83±31.62	68.64±34.83	87.19± 46.30
Easy minutes	201.87 ± 57.71	184.38± 82.72	208.17± 50.91	198.51± 90.18	186.26± 67.44	150.82 ± 100.52
Very easy minutes	549.66 ± 178.31	511.85 ± 138.37	562.34 ± 184.42	549.57 ± 157.32	593.37 ± 147.68	669.09 ± 288.42

INT: Intervention group; PAQ: Physical Activity Questionnaire; HFS worry: Fear of Hypoglycaemia Survey, worry subscale HFS do: Fear of Hypoglycaemia Survey, action subscale; *CSAPPA* Children’s Self-perception of Adequacy in and Predilection for Physical Activity questionnaire, *CHU9D* Child’s Health Utility form, *CHQ* Child Health Questionnaire; HbA1C low knowledge: Accuracy of parent’s knowledge of the lowest acceptable value for HbA1c; HbA1c high knowledge: Accuracy of parent’s knowledge of the highest acceptable value for HbA1c.

Audio recordings of qualitative interviews were transcribed verbatim and analysed using the framework approach [21]. Three main themes were focused upon: comprehending medical/health research; suggestions for and reflections on SKIP research processes; and, comments on the STAK-D intervention.

## Results

### Usual care

Both sites reported comparable usual care responses. Overall usual care score was similar between sites at each time-point and did not change over time. Both clinics reported usual clinics to take place every three months. Additional services reported by NUH were: family therapy, social services referrals, mixed gender therapy group, youth club, residential retreats, formal education clinic and ad hoc information as required. Additional services reported by UHL were: psychiatric/mental health service, social service, peer support group, parent group, a coeliac website, YouTube and a clinic developed smartphone application.

### Feasibility assessment

#### Recruitment

The two sites identified a total of 142 (58 NUH and 84 UHL) eligible patients at the beginning of the study. All were sent postal invitations to SKIP by their clinical team, and 85 (60%) of these were subsequently approached face-to-face in clinic. An additional 12 participants were identified to have become eligible in the second year of the study and were approached in clinic. This led to 56 written expressions of interest being received (36% of 154 eligible participants), from which 49 participants (88%) consented and enrolled into SKIP. The main reason for not recruiting after receipt of an expression of interest was that the patient was not contactable. In interviews, participants reported that the randomisation process was acceptable with 24 allocated to the intervention group and 25 to the control group. There was little difference in most demographic and clinical characteristics between the groups, though BMI was higher and father’s education lower in the intervention group (Table 3). A total of 41 participants were recruited face-to-face in clinic, relative to eight being recruited following returned postal expressions of interest. Response rate to posted SKIP invitations was 10.6% with 33.3% of these refusing (3.5% of those sent a postal invite). Refusal rate to in-clinic approaches was 42.3%. The main reasons for refusing participation were ‘not being interested’, ‘wanting more time to read the information pack at home’ (and then not returning the invitation slip) and it ‘not being the right time’.

**Table 3:** Demographic and clinical characteristics of SKIP participants according to group allocation

Demographic or clinical characteristics	Control (*N*=25)	Intervention (*N*=24)	All
Mean age (SD)	10.89 (0.9)	10.40 (1.1)	10.63 (1.0)
BMI (kg/m^2^)	17.09 (2.0)	20.69 (3.6)	18.98 (3.4)
Basal insulin dose (units)	11.06 (6.1)	12.30 (6.3)	11.71 (6.1)
Bolus insulin dose (units)	18.18 (9.4)	23.69 (20.6)	21.08 (16.3)
	N (%)	N (%)	N (%)
Site			
NUH	13 (52.0)	15 (62.5)	28 (57.1)
UHL	12 (48.0)	9 (37.5)	21 (42.9)
Gender			
Female	8 (32.0)	14 (58.3)	22 (44.9)
Male	17 (68.0)	10 (41.7)	27 (55.1)
Ethnic origin			
White	22 (88.0)	20 (83.3)	42 (85.7)
Black British	0 (0.0)	1 (4.2)	1 (2.0)
Asian	1 (4.0)	1 (4.2)	2 (4.1)
Mixed race	1 (4.0)	2 8.3)	3 (6.1)
Other	1 (4.0)	0 (0.0)	1 (2.0)
Insulin delivery method			
Multiple daily injections	7 (29.2)	7 (30.4)	14 (29.8)
Insulin pump	16 (66.7)	15 (65.2)	31 (66.0)
Insulin pen	1 (4.2)	1 (4.3)	2 (4.3)
Method of glucose monitoring			
Self-monitoring	20 (83.3)	18 (78.3)	38 (80.9)
Continuous glucose monitoring system	4 (16.7)	5 (21.7)	9 (19.1)
Mother’s attributes			
Lives with mother	23 (95.8)	22 (95.7)	45 (95.7)
Mother employed	17 (68.0)	18 (78.3)	35 (72.9)
Mother without formal educational qualifications	4 (16.0)	2 (10.0)	6 (13.3)
Father’s attributes			
Lives with father	23 (95.8)	17 (73.9)	40 (85.1)
Father employed	20 (83.3)	18 (81.8)	38 (82.6)
Father without formal educational qualifications	2 (8.0)	5 (23.8)	7 (15.2)
Family income			
Less than £20,000	3 (13.0)	6 (27.2)	9 (20.0)
£20,000-£40,000	8 (34.7)	7 (31.8)	15 (33.4)
More than £40,000	11 (47.8)	9 (40.9)	20 (44.4)

### Engagement with STAK-D and watch synchronising

Number of visits per week to STAK-D pages averaged 12.37 at T0, 4.31 between T0 and T1, and 0.55 between T1 and T2, respectively. Downloads per week of the goal sheet and goal certificate respectively averaged 0.14 and 0.00 at T0, 0.01 and 0.02 at T1, and 0.00 and 0.00 at T2. The ‘Kids Zone’ was the most visited page. Participants synchronised their watches on 48 out of the required 144 occasions (33.3%; weekly, weeks one-seven of intervention).

### Retention and measure completion rate

At T1 and T2 respectively, 75.5% and 77.6% of participants were met to collect follow-up data. Completion rate for all variables is given in Table 4. All non-clinical variables at all three time-points met the aim of >70% completion to be judged as acceptable. All non-clinical variables also met the aim of >85% completion to be judged as feasible, except for clinician communication (79.6%) and objectively measured physical activity (78.9%) which were both marginally short of the feasibility target at T2. Complete clinical data (HbA1c, BMI, insulin) was available for 63.3% to 63.5% of participants at each data collection time-point, falling short of both feasibility and acceptability targets. However, all children had valid HbA1c data at all time-points. BMI was calculated for 98% of children (*n*=48) at T0, 93.9% of children (*n*=46) at T1 and 83.7% of children (*n*=41) at T2. Insulin dosage was provided for 73.5% of children (*n*=36) at baseline, 75.5% of children (*n*=37) at T1 and 69.4% of children (*n*=34) at T2. There were no significant differences in demographics or baseline responses between those completing eight week and six-month follow-ups and those who did not.

**Table 4:** Completion of individual scales at baseline, eight week follow-up and six month follow-up

	Baseline completion (*N*=49), n (%)	Eight week completion (*N*=37), n (%)	Six month completion (*N*=38), n (%)
Child measures			
Communication	49 (100)	37 (100)	38 (100)
PAQ	48 (98.0)	37 (100)	36 (94.7)
HFS	45 (91.8)	33 (89.2)	36 (94.7)
CSAPPA	48 (98.0)	35 (94.6)	37 (97.4)
CHU9D	48 (98.0)	37 (100.0)	38 (100)
Burden	48 (98.0)	37 (100.0)	37 (97.4)
Clinical record (*N*=49)	32 (65.3)	31 (63.3)	31 (63.3)
Parent measures			
Baseline	47 (95.9)	37 (100)	37 (97.4)
CHQ	45 (91.8)	34 (91.9)	38 (100)
HFS	48 (98)	35 (94.6)	36 (94.7)
Burden	47 (95.9)	37 (100.0)	37 (94.7)
Clinician measures			
Communication^a^	49 (100)	43 (87.8)	39 (79.6)
Objective physical activity			
at least 600 minutes ≥ three days/week	43 (87.8)	33 (89.2)	30 (78.9)

*PAQ* Physical activity questionnaire, *HFS* Hypoglycaemia fear survey, *CSAPPA* Children’s self-perceptions of adequacy in and predilection for physical activity scale, CHU9D Children’s healthy utility scale, *CHQ* Children’s health questionnaires. ^a^*N*=49 for baseline, eight week and six month follow-ups

### Participant burden and satisfaction

At all time-points the majority of participants (T0: 57.1%; T1: 94.6%; T2: 83.8%) and parents (T0: 61.2%; T1: 81.1%; T2: 81.1%) reported completing the questionnaires within 0-20 minutes. The proportion of children completing within ten minutes increased over time from 16.3%, to 30.6%, to 40.8%. All participants reported understanding all questions; however, 14.3% to 16.3% of children reported finding one or more questions too difficult to answer at each time-point. Of the children, 59.2% and 48.6% reported needing help to answer questions at T0 and T1 respectively. This dropped to 40.8% at T2 (six months). Only 20.4% of parents reported needing help at baseline, reducing to 10.2% at T1 and T2 follow-ups.

There were 82.4% of participants and 87.5% of their parents reporting that they enjoyed being part of the STAK-D programme. In addition 29.4% and 50.0%, and 23.5% and 18.8% of children and parents respectively, reported learning something new about physical activity and changing the amount of physical activity they do. The PAW was the most popular component of the intervention with 47.1% reporting liking the PAW ‘a lot’, relative to 35.3% liking the STAK-D website ‘a lot’.

### Fidelity and safety

One intervention participant was unable to download PAW synchronisation software on their home computer, even after an additional home visit by a project researcher involving calls to the Polar helpline, to resolve the issue.

In the end of study questionnaire (T2) all parents reported access of their child to the internet at home both retrospectively (prior to SKIP start) and prospectively (at SKIP end). All parents stated that their child had access to the internet at home. Access at school prior to SKIP was reported by 87.5% of parents, with 93.3% of parents reporting their child to have access to the internet at school by SKIP end. Daily use, both before and after SKIP, of personal computers, tablets and smartphones, was reported by 37.5%, 56.3% and 50.0% of parents, respectively. According to their parents 12.5% of children never used personal computers before or after SKIP. Wearable devices were the only technological devices for which children’s use was reported by their parents to be different prior to and after the intervention, with usage increasing from 0.0% to 25.0%.

All intervention group participants were sent a minimum of three prompts a week to synchronise their PAW, between weeks two and seven of the intervention. Despite this, lack of response and failure of participants to synchronise their PAW meant that personalised feedback reports were sent on only 48 of 144 occasions (33.3%). More participants were recruited from one of the participating sites. Recruitment processes were initially different between sites due to a difference in interpretation of the protocol at each site. Two changes were made to the protocol during the intervention; one of these changes was to address this disparity in interpretation, the other related to the process for the collection of qualitative data. No adverse events were reported by participants or wider project staff.

### Between-group outcomes

Table 5 provides full results from the multi-level modelling analysis. Two variables produced significantly different change scores from T0 to T1 follow-up between control and intervention groups. The PAQ sedentary scale decreased (improved) in the intervention group relative to the control group (-2.28; p<0.05; 95% CI = -4.40, -0.16; p = 0.04; *d*_*ppc2*_ = 0.72). The CHQ physical t-score increased in the intervention group relative to the control group (6.15; p<0.05; 95% CI = 1.75, 10.55; p = 0.01; *d*_*ppc2*_ = 0.75). No variables produced significantly different change scores from T0 to T2 follow-up between control and intervention groups.

**Table 5:** Results from the multi-level modelling analysis

	Baseline to eight week follow-up				Baseline to six month follow-up			
	Control	Intervention	Comparison		Control	Intervention	Comparison	
	Mean ∆ (95% CI)	Mean ∆ (95% CI)	Mean ∆ ^mcscba^ (95% CI)	p	Mean ∆ (95% CI)	Mean ∆ (95% CI)	Mean ∆^mcscba^ (95% CI)	p
Insulin dose (basal)	0.74 (-0.18, 1.66)	0.84 (-0.08, 1.77)	0.11 (1.20, 1.41)	0.87	1.01 (-0.10, 2.11)	1.64 (0.61, 2.67)	0.63 (-0.89, 2.14)	0.42
Insulin dose (bolus)	2.57 (0.14, 5.00)	1.44 (-1.01, 3.89)	-1.13 (-4.60, 2.34)	0.52	3.03 (0.48, 5.58)	5.21 (2.70, 7.72)	2.18 (-1.43, 5.79)	0.24
HbA1c	0.46 (-1.94, 2.86)	1.91 (-0.54, 4.36)	1.45 (-1.98, 4.88)	0.41	1.14 (-1.26, 3.54)	2.85 (0.40, 5.30)	1.71 (-1.72, 5.14)	0.33
BMI	0.01 (-0.31, 0.33)	0.45 (0.12, 0.77)	0.44 (-0.02, 0.91)	0.06	0.14 (-0.21, 0.48)	0.36 (0.02, 0.70)	0.22 (-0.27, 0.71)	0.38
PAQ physical activity	0.21 (-3.91, 4.33)	-0.88 (-5.97, 4.21)	-1.09 (-7.64, 5.46)	0.75	0.74 (-3.46, 4.95)	0.41 (-4.60, 5.42)	-0.33 (-9.87, 6.21)	0.92
PAQ sedentary	0.18 (-1.13, 1.49)	-2.10 (-3.77, -0.44)	-2.28 (-4.40, -0.16)	0.04	-0.22 (-1.58, 1.14)	-0.52 (-2.14, 1.10)	-0.30 (-2.42, 1.82)	0.78
Frequency of after-school clubs	-0.12 (-1.75, 1.51)	0.27 (-1.81, 2.36)	0.40 (-2.26, 3.05)	0.77	-0.22 (-1.92, 1.48)	-0.06 (-2.01, 1.89)	0.16 (-2.43, 2.75)	0.91
Steps per day	540.21 (-799.26, 1879.68)	1701.75 (-140.17, 3543.66)	1161.54 (-1118.20, 3441.27)	0.32	23.06 (-1348.02, 1394.14)	921.26 (-1104.70, 2947.22)	898.20 (-1551.48, 3347.88)	0.47
Easy minutes per day	10.00 (-12.26, 32.26)	-1.79 (-33.50, 29.92)	-11.78 (-50.53, 29.96)	0.55	-17.38 (-40.08, 5.32)	-45.34 (-78.54, -12.14)	-27.96 (-68.22, 12.30)	0.17
Moderate minutes per day	0.39 (-7.01, 7.79)	7.56 (-2.46, 17.58)	7.17 (-5.29, 19.63)	0.26	-3.52 (-11.05, 4.02)	3.86 (-7.00, 14.71)	7.37 (-5.84, 20.58)	0.27
Vigorous minutes per day	0.13 (-5.53, 5.78)	-0.76 (-8.94, 7.43)	-0.88 (-10.83, 9.07)	0.86	-2.10 (-7.90, 3.69)	6.53 (-2.08, 15.14)	8.64 (-1.74, 19.01)	0.10
CSAPPA total score	0.18 (-2.37, 2.74)	-1.94 (-5.15, 1.27)	-2.13 (-6.26, 2.01)	0.31	-0.42 (-3.05, 2.21)	-1.64 (-4.71, 1.43)	-1.22 (-5.29, 2.86)	0.56
CSAPPA adequacy score	-0.33 (-1.63, 0.97)	-0.40 (-2.05, 1.25)	-0.06 (-2.19, 2.06)	0.95	-0.49 (-1.83, 0.86)	-0.53 (-2.09, 1.03)	-0.05 (-2.13, 2.04)	1.00
CSAPPA predilection score	4.20 (2.57, 5.83)	3.00 (0.98, 5.02)	-1.20 (-3.80, 1.41)	0.37	3.55 (1.88, 5.21)	3.27 (1.31, 5.22)	-0.28 (-2.86, 2.30)	0.83
CSAPPA enjoyment score	0.11 (-0.44, 0.66)	-0.19 (-0.88, 0.51)	-0.30 (-1.19, 0.59)	0.51	0.13 (-0.43, 0.70)	0.03 (-0.64, 0.69)	-0.11 (-0.99, 0.77)	0.81
CHU total score	0.51 (-0.80, 1.83)	0.52 (-1.11, 2.16)	0.01 (-2.09, 2.11)	0.99	0.57 (-0.77, 1.92)	2.01 (0.44, 3.58)	1.44 (-0.63, 3.50)	0.17
Child HFS total score	-6.02 (-10.04, -1.99)	-2.10 (-7.21, 3.00)	3.91 (-2.60, 10.42)	0.24	-3.49 (-7.75, 0.78)	-5.l7 (-10.00, -0.35)	-1.68 (-8.12, 4.76)	0.61
Child HFS worry score	-3.86 (-7.47, -0.26)	-2.13 (-6.69, 2.43)	1.73 (-4.09, 7.56)	0.56	-3.03 (-6.83, 0.78)	-4.36 (-8.67, -0.04)	-1.33 (-7.09, 4.43)	0.65
Child HFS do score	-2.27 (-4.35, -0.20)	0.12 (-2.52, 2.76)	2.39 (-0.96, 5.75)	0.16	-0.40 (-2.56, 1.75)	-0.84 (-3.33, 1.65)	-0.44 (-3.73, 2.86)	0.80
Parent HFS total score	0.24 (-4.95, 5.43)	-0.68 (-7.39, 6.04)	-0.91 (-9.40, 7.57)	0.83	-4.58 (-9.91, -0.76)	-5.61 (-12.03, 0.82)	-1.03 (-9.38, 7.32)	0.81
Parent HFS worry score	-1.60 (-5.95, 2.75)	-2.13 (-7.78, 3.51)	-0.53 (-7.66, 6.59)	0.88	-4.53 (-9.01, -0.05)	-4.32 (-9.70, 1.07)	0.21 (-6.79, 7.22)	0.95
Parent HFS do score	1.93 (0.27, 3.60)	1.36 (-0.84, 3.56)	-0.58 (-3.34, 2.18)	0.68	-0.11 (-1.84, 1.62)	-1.44 (-3.50, 0.62)	-1.33 (-4.03, 1.36)	0.33
CHQ physical T-score	-2.17 (-4.91, 0.57)	3.98 (0.54, 7.42)	6.15 (1.75, 10.55)	0.01*	-0.33 (-3.15, 2.50)	2.10 (-1.18, 5.38)	2.43 (-1.91, 6.76)	0.27
CHQ psychological T-score	-0.85 (-3.95, 2.26)	-1.01 (-4.94, 2.91)	-0.17 (-5.17, 4.84)	0.95	1.72 (-1.49, 4.94)	-1.46 (-5.18, 2.26)	-3.18 (-8.10, 1.74)	0.21
School days missed by child	-1.90 (-2.41, -1.39)	-1.41 (-2.05, -0.77)	0.49 (-0.34, 1.31)	0.25	-2.05 (-2.57, -1.52)	-1.33 (-1.95, -0.71)	0.72 (-0.09, 1.54)	0.08
HbA1c aim low accuracy	0.02 (-0.18, 0.22)	0.14 (-0.12, 0.39)	0.12 (-0.21, 0.44)	0.49	0.12 (-0.09, 0.33)	0.08 (-0.16, 0.32)	-0.04 (-0.36, 0.28)	0.81
HbA1c aim high accuracy	0.18 (-0.74, 1.10)	0.36 (-0.77, 1.49)	0.18 (-1.28, 1.64)	0.81	0.40 (-0.53, 1.33)	0.08 (-1.02, 1.18)	-0.32 (-1.77, 1.13)	0.66
Clinician communication score	1.56 (0.51, 2.60)	2.38 (1.23, 3.53)	0.83 (-0.73, 2.38)	0.30	2.17 (1.09, 3.24)	2.92 (1.73, 4.11)	0.76 (-0.85, 2.36)	0.36

∆: change score; 95% CI: 95% confidence interval; ∆^mcscba^: modelled change score comparison between arms

*INT* Intervention group, *PAQ* Physical Activity Questionnaire; HFS worry: Fear of Hypoglycaemia Survey, worry subscale HFS do: Fear of Hypoglycaemia Survey, action subscale, *CSAPPA* Children’s Self-perception of Adequacy in and Predilection for Physical Activity questionnaire, *CHU9D* Child’s Health Utility form, *CHQ* Child Health Questionnaire, *HbA1C low knowledge* Accuracy of parent’s knowledge of the lowest acceptable value for HbA1c; HbA1c high knowledge: Accuracy of parent’s knowledge of the highest acceptable value for HbA1c

### Qualitative analysis

Eight HCPs and eight parent-child dyads were interviewed. Data were handled using the NVivo software package and organised using Framework analysis.

#### Comprehending medical/health research

Both HCPs and child-parent dyads considered research in healthcare to be a positive. HCPs cited improvements to the child’s health, as well as to the health of the entire family. Child-parent dyads referred to research as *establishing a proof of concept*, in *advancing healthcare* or in *benefitting others*. Families recognised that research might be a way of *being part of something important* and *connecting with others*; this could be other children with T1DM and their families, or other healthcare staff.

*“anything that comes, um especially [child] to contribute then we’re always keen to do that whatever, if anything can help others then it’s always a good thing to do and [child’s] keen to do it as well”* (C44, parent)

There was some disagreement in the HCP responses about whether healthcare research requires positive findings to be of use; some indicated that the process of research alone might be beneficial independent of any substantive outcome.

HCPs described a very high level of research activity at their respective sites. This was a positive, but was also identified as bringing with it a high workload and generating the potential for study overload and apathy in the patient group – although no family indicated this. Further, while HCPs considered research as an aid to healthcare delivery, it was suggested that not all members of the healthcare team valued it highly and that research sometimes failed to engage those individuals and families that might benefit most from participation.

*“we tend to get a cohort of uh families who engage in research who seem to be the same sort of cohort so the difficult to access ones are the ones that we will find difficult to consent”* (HCP 2)

#### Suggestions for and reflections on SKIP research processes

A number of benefits for taking part in SKIP were identified with HCPs mentioning that it helped them to learn more about research processes and share responsibility for physical activity promotion with a wider team. Parents and children enjoyed the opportunity to involve more family members in discussions around the child’s care. With regards to research processes, parents and children found involvement as presenting little burden. Face-to-face recruitment was identified as crucial to persuading participation by dyads and HCPs alike. Some HCPs did mention experiencing time pressures and being uncertain of some expectations, however, all remained positive about participation in future research and reported improvements to practices within the clinic around recruitment.

*“I think that’s actually what SKIP’s done. It tended to be one of the tick boxes in clinic and actually we’ve gained more focus towards talking about exercise, in fact it’s got us thinking about what education we’re going to deliver about exercise in the future”* (HCP 2)

The biggest challenge identified by both HCPs and dyads related to the chosen technology. It is unsurprising that both children and parents reported children losing interest in the research when they lost or experienced technical difficulties with their PAW. Other reported reasons for the child losing interest included the website not appealing or not appearing interesting to them, the website containing too much information, website mechanics being clunky and discomfort experienced from the PAW.

*“When it [the watch] was waterproof I always had it on so I left it on to sleep, sometimes I took it off. But then I wouldn’t have to put it on cos it was already there. Now that it’s not waterproof I like forget to put it back on after a shower”* (C19 child)

#### Comments about the STAK-D intervention

Every child interviewed reported becoming more physically active at some point during the course of their involvement in SKIP, though this was often only for the short-term. Receiving activity reports from the PAW was a positive element of the STAK-D programme and was influential in forming positive habits.

*“So we knew [due to the feedback report] we needed to go out a bit more on a Sunday, which we do actually do quite a bit now so”* (C24 child)

Interviews exposed a range of competition for the STAK-D programme that may have discouraged active engagement; these included available alternative systems which targeted similar behaviours, competition from commercially available alternative monitors, and support or information from other sources (e.g. friends, family) which rendered STAK-D unnecessary.

*“because there’s the sport clinic now that they’ve just started… that would be a way easier way of engaging in exercise for patients cos there’s always that fear of having a hypo um, and you’ve got trained staff around to make sure that that doesn’t happen. Whereas the STAK-D it’s like you’re kind of having to input without like a reward or anything”* (HCP 6)

A number of suggestions were made to improve STAK-D: rewards, improved technological functionality, automaticity of feedback, greater family involvement and greater variety of content.

*“I think the best inbetweener would be the app yeah but then if its automated as well that would be even more so but the app even more so because you could enter whatever you wanted to and its instantly logged and then that’ll go a long way, especially with children and young adults cos its that, that instant communication its almost paramount as opposed to waiting till you get home”* (C44 parent)

## Discussion

The SKIP study aimed to assess the feasibility and acceptability of a RCT of a website-based physical activity and self-monitoring programme (STAK-D) in children with T1DM. This research addresses a gap in the evidence-base around technology-based interventions to promote self-management behaviours in children with T1DM [9]. Our results indicate that SKIP recruitment and data collection processes were acceptable as most objective targets were met and participants reported the burden imposed by completion of questionnaires and wearing a PAW to be low. Further, all children interviewed indicated that the randomisation process was acceptable. This indicates that the SKIP protocol was effective in engaging young people with T1DM and their parents in research studies. Given the child’s clinic was the key setting, there is reason to believe that similar approaches could be used to increase engagement with research in other paediatric populations with chronic conditions [22, 23]. Although the completion rate for full clinical data fell below pre-determined acceptability and feasibility targets at all time-points, completion rates were in line with other similar studies [24, 25]. The challenges for clinical data collection were largely related to accessing records for insulin dose, whereas collection of HbA1c and calculation of BMI was less problematic. Whilst the objective must remain to collect data as completely as possible, the stringent targets used in the present study may have been ambitious for elements of the clinical data.

Compliance with PAW wearing was lower than for self-reported data, but was not far away from our pre-determined criteria for acceptability. Previous intervention studies with children have reported lower completion rates for objectively measured physical activity. For example, completion rates of 60% [26], 54-42% [27] and 41-74% [28] across follow-up time-points, have recently been reported in physical activity intervention studies with children. Another study has reported higher completion rates for objectively measured physical activity (85%), though a courier service was used which may have boosted response [29]. Findings from the present study suggest that physical activity data collection using a simple activity wristwatch is likely to be feasible if technical problems are avoided and effective strategies to prompt re-engagement are identified.

The SKIP project incorporated the STAK programme [11] in the form of a website designed for children with T1DM (STAK-D), including components identified as helping individuals change their behaviours, such as physical activity goal setting, planning and self-monitoring [30]. However, engagement with STAK-D was low with children logging in infrequently and parents barely logging in at all. Interest in STAK-D was highest in the first days of involvement, with participants becoming disinterested shortly afterwards. There are a number of explanations for this. Firstly, as has been uncovered in national trends [7], few participants regularly used personal computers, preferring to access the internet on tablets or mobile phones. Whilst STAK-D could be used on other devices, viewing and functionality were sub-optimal on these devices as it was designed to be accessed on a desktop computer, a next step should be to develop a user-friendly STAK-D interface for use on other devices. Secondly, parents and children described the STAK-D website as containing too much information, and not being user-friendly. Enjoyment is crucial for promoting physical activity in care settings [31] and encouraging young people to engage with interventions [32]. Thirdly, effective strategies of increasing engagement through feedback and rewards [30] were not delivered as intended because they were reliant on initial participant input. Only one third of watch synchronisations were performed by participants as intended, characterised by a few participants completing most of their synchronisations and the majority of participants completing few or no synchronisations independently. Objective measures with automatic synchronisation options should be investigated when intervention fidelity requires the ongoing receipt of participant data. Finally, a number of technical problems with the site were reported such as, password failure and glitches. While these incidents were temporary and infrequent, it is clear that small glitches in technological tools can interfere with its use and deter future engagements. Technology-based interventions pose a great challenge as the speed with which what is ‘novel’ changes and exceeds the parameters within which research normally operates. With wearable technology (including smartphones and activity trackers) being one of the fastest growing technology markets 2015-2019 [33], research challenges will only increase. Further, as availability of technology to children increases [7], children will continue to expect more from the technology they use and future research studies must contend with this. Also, parents of children with T1DM continue to express positive views towards the potential of digital resources in diabetes care [34].

Despite low engagement with STAK-D and some technical issues with participant wristwatches, short-term improvements were found in the intervention group. Children reported engaging in less sedentary behaviour, while parents reported perceiving their children to be in better physical health, at eight-week follow-up. As SKIP did not explicitly target sedentary behaviour, it is interesting that children reported less sedentary behaviour but not more physical activity. Given anecdotal evidence that the PAWs alerted both children and parents to periods when they were typically inactive, it is likely that this prompted sporadic activity to break up these periods rather than guideline fulfilling physical activity. This is supported by the lack of change in objectively measured moderate-to-vigorous physical activity. It is interesting that parents perceived an improvement in their child’s physical health and not in their psychological health. Previous research does not suggest that the physical variable is more amenable to change [35, 36]. A more likely explanation is indicated by qualitative findings that parents of children exposed to the intervention became more conscious of their child’s overall physical activity levels. This was also the case for children who did not become more active, with their parents stating that they had become more aware of how active their child already was. It should be noted that the present study predominantly attracted already active children to participate, although this is a common problem in physical activity research [37]. However, reducing sedentary behaviour has health benefits which are independent from physical activity behaviour [38]. Whilst future physical activity research should seek to find ways of reaching hard-to-reach inactive individuals, future iterations of SKIP should also seek to maximise effects on sedentary behaviour.

As per protocol [10], following the eight-week follow-up visit (T1), aspects of the intervention were discontinued including weekly prompts to wear the PAW and login to STAK-D, and weekly personalised activity reports (since these were available for an eight week intervention period). This could at least partially explain why beneficial effects observed from baseline to eight weeks did not persist at six months. As has been previously recommended by a systematic review of computer and internet-based interventions in children, the present research suggests that maintenance efforts are necessary to ensure long-term positive effects [39]. Further, a recent systematic review highlights that to produce clinically relevant outcomes, researchers might consider targeting technology-based interventions to those individuals who demonstrate poor diabetes management [40].

### Limitations

Several limitations were identified in the study. Although we were able to collect reasons for non-participation, we were not able to collect data on the demographic or clinical characteristics of those who declined participation. The PAW was not designed specifically for research purposes, however, it was selected since it has a comfortable, waterproof watch-style design and digital display features, provided instant feedback, and was shown to be better accepted by children compared to other research-grade monitors [41]. The intervention was focused primarily on physical activity rather than overall self-management of T1DM; nevertheless, the educational content provided within STAK-D included self-management and wellbeing approaches, and provided guidance on the importance of regular blood glucose monitoring, and managing physical activity around diet. The sample was small, limiting the conclusions that can be drawn, although it was sufficient to address our feasibility and acceptability aims. Further, ethnic minority groups are under-represented and participants were over-recruited from one site, which limits generalisability.

## Conclusions

This study investigated the feasibility and acceptability of undertaking a RCT of an internet-based physical activity programme (STAK-D) to enhance self-efficacy and self-monitoring in children with T1DM. Although evidence of long-term effects could not be reported, key elements of feasibility and acceptability were identified. Results demonstrated reasonable demand for SKIP, successful intervention delivery within the desired population, research processes which were practical for participants and staff, and evidence of short-term efficacy in some outcomes. Further exploration of the intervention is both required and justified to refine and understand its components and enhance its capacity to create measurable and long lasting change.

## Additional files

Additional file 1: CONSORT Checklist. CONSORT Checklist for reporting of randomised controlled trials. (DOCX 48 kb)

Additional file 2: Participant Interview Guide. Interview questions for participants in the intervention group, to evaluate perceptions of the research process, usage of STAK-D, satisfaction, accessibility, effectiveness, facilitators of and barriers to change. (DOCX 42 kb)

## Abbreviations

CHQ: Child’s Health Questionnaire
CHU-9D: Perceived Health Questionnaire
CI: Confidence interval
CSAPPA: Predilection for Physical Activity Questionnaire
HbA1c: glycosylated haemoglobin
HCP: Healthcare professional
HFS: Fear of hypoglycaemia survey
NUH: Nottingham University Hospitals NHS Trust
PAQ: Physical Activity Questionnaire
RCT: Randomised controlled trial
SD: Standard deviation
SKIP: Supporting Kids with Diabetes in Physical activity
STAK-D: Steps To Active Kids with Diabetes
T0: baseline
T1: eight week follow-up
T1DM: Type 1 diabetes mellitus
T2: six month follow-up
UHL: University Hospitals of Leicester NHS Trust
