# Supplementary material for: SKIP (Supporting Kids with diabetes In Physical activity): Feasibility of a randomised controlled trial of a digital intervention for 9-12 year olds with type 1 diabetes mellitus
Source: BMC Public Health. 2019 Apr 3;19:371. doi: 10.1186/s12889-019-6697-1 (PMC6446303; doi:10.1186/s12889-019-6697-1)
Supplement: Supplementary file 2 — Participant Interview Guide. Interview questions for participants in the intervention group, to evaluate perceptions of the research process, usage of STAK-D, satisfaction, accessibility, effectiveness, facilitators of and barriers to change. (DOCX 42 kb) [file 12889_2019_6697_MOESM2_ESM.docx]

**
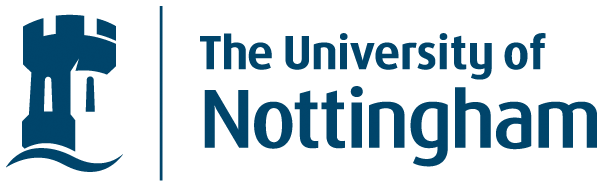
**

**University of Nottingham, School of Health Sciences**

# Supporting Kids with diabetes in Physical activity:

# An online multimedia intervention to promote physical activity in children with type 1 diabetes (T1DM)

**Interview guide**

• General introduction / ice breaker

• Recap purpose of the interview

- I’d like to ask you about what you thought about the STAK programme. Please be honest.
- The interview will be recorded, with permission, and then I will type it all up to use as data for my research.
- If there’s any questions you do not want to answer then that’s ok and if at any time you want the interview to stop that’s fine too.
- If it’s ok with you I will turn the recorder on now and everything we talk about from now on will be recorded.
- Show child/children the Dictaphone.
- *Are you happy for me to turn the tape on?*

**Start recording**

***** Obtain verbal consent to participate on tape *****

You provided consent at the very beginning of this study, but this does not mean you have to participate in this interview.

*Are you happy to continue with the interview?*

**Research evaluation**

These questions are about the research study

- What did you think about how you were recruited?
  - Receiving a letter at home – did you receive it/read it/respond to it? Why?
  - How did you first see or hear about Skip
  - What interested you and led you to express interest?
  - Did you see any other information about Skip anywhere? e.g. clinic posters, website, at a group?
- What did you like about being involved in this research?
  - How did you find filling in the questionnaires?
  - What did you think about us choosing children at random to be in the STAK-D group?
  - Were you happy with the group you were put into?
  - Would you recommend this research to other children with Type 1 Diabetes?
- *Parent:* Would you like to be offered a physical activity programme as part of your child’s usual care? (advice and guidance around physical activity).
- *Child:* Would you like to hear more about physical activity in clinic? What sort of information would you like?

**Usage**

- How much did you use the STAK-D programme?

Did not use it Once or twice but no more Once a week Every day

- - Can you explain why you used it this much?

**User-satisfaction with the STAK-D programme**

- What did you think about the STAK-D programme?
  - Prompt *enjoyment*.
- Explore individual components:
  - Website
    - How easy was it to use?
    - How interesting was it?
    - How useful was it?
    - What parts of the website did you like the most?
    - What parts did you use the most?
    - Will you continue to go on the website?
    - Do you understand what I mean if I talk about the 5-a-day Skip activity goal. Did you think 5 pieces of activity was realistic?
    - Did you set any goals? What goals did you set yourself?
      - How useful were the goals you set yourself?

If child didn’t use: Why did you not use the website?

- - - Did you login in the beginning?
    - Do you visit other websites related to Diabetes or physical activity?
    - How might we have encouraged you to use the website?
    - Did the website meet your expectations from when the study was first explained to you and you consented to take part in the study?
    - If it was the right one – do you think you would ever use a website to find information on diabetes or physical activity?

Activity Reports

- Did you share them with your child?

- Were they useful?

- Did they meet expectations?

- - Activity monitor
    - Were you excited to receive the watch at the beginning of the study?
    - Do you think the watch is a good idea? What feature?
    - How easy was the watch to wear/remember to wear?
    - What did you like/not like about the watch?
    - Did you wear the watch even when you weren’t being reminded/measured? What would have encouraged you to wear it more often?
    - Did you find the activity reports useful? Why? What would you have liked to have seen?
    - Did you have any problems with your watch? Did this influence how you felt about wearing it?
- What did you enjoy least about the study?
  - Why?
- How would you make the STAK-D programme better?
  - What would you like to see on the website?
- Would you recommend the STAK-D programme to other children with Type 1 Diabetes?

**Accessibility**

- How was it for you to access the website?
  - Prompt availability of internet/computer/smart phone
  - Did anything ever go wrong with the website
- How was it for you/your child to use the activity watch?
  - Prompt how easy it was to use.
  - Did anything ever go wrong with the watch?

**Effectiveness**

- How did STAK-D, wearing the watch or any other aspect of the study, make you feel?
- Did you notice any changes in yourself?
  - Prompt: How did it change your activity level?
  - Why do you think those changes happened? / didn’t happen?
- *If appropriate:* How did being more active make you feel?
  - Were there any side-effects of being more active?
  - Do you think the effects [repeat what they said, if appropriate] will continue?
- Did your family get involved?
  - How did that make you feel?
- Did your friends get involved?
  - How did that make you feel?

**Facilitators of/Barriers to change**

- What made it easy to be active?
  - Prompt: did family or friends help?
- What made it hard to be active?
  - What things got in the way?
- Is there anything else you would like to say about the STAK-D programme?
- Do you have any questions for me?
- Do you have any concerns or queries before I turn off the tape?

Thank you for answering these questions.

** TURN OFF THE DICTAPHONE **

**END OF INTERVIEW.**
